# COPB1-knockdown induced type I interferon signaling activation inhibits *Chlamydia psittaci* intracellular proliferation

**DOI:** 10.3389/fmicb.2025.1566239

**Published:** 2025-03-06

**Authors:** Nana Li, Huiying Yang, Shan Zhang, Yufei Jiang, Yinhui Lin, Xiaoxiao Chen, Yuchen Zhang, Yonghui Yu, Xuan Ouyang, Yujun Cui, Yajun Song, Jun Jiao

**Affiliations:** ^1^Department of Epidemiology and Health Statistics, School of Public Health, Anhui Medical University, Hefei, China; ^2^State Key Laboratory of Pathogen and Biosecurity, Academy of Military Medicine Sciences, Beijing, China; ^3^School of Public Health, Mudanjiang Medical University, Mudanjiang, Heilongjiang, China

**Keywords:** *Chlamydia psittaci*, COPB1, type I interferon, STING, Golgi

## Abstract

**Objective:**

*Chlamydia psittaci* is a zoonotic pathogen that causes an acute disease known as psittacosis. To establish infection in host cells, *Chlamydia* manipulates the host cell’s membrane trafficking pathways.

**Methods:**

In this study, using fluorescently labeled *C. psittaci* and screening a human membrane trafficking small interfering RNA (siRNA) library, we identified 34 host proteins that influenced *C. psittaci* infection in HeLa cells.

**Results:**

Among these, knockdown (KD) of two genes encoding subunits of the coatomer complex I (COPI) inhibited the pathogen’s intracellular survival. Specifically, the knockdown of COPB1, a COPI subunit, significantly reduced the intracellular proliferation of *C. psittaci*. Mechanistically, we found that type I interferon negatively affected *C. psittaci* infection. Moreover, COPB1 KD disrupted the homeostasis of STING, preventing its retrieval from the Golgi back to the endoplasmic reticulum (ER), which in turn activated type I interferon signaling.

**Conclusion:**

Together, our findings advance the understanding of the mechanisms underlying *Chlamydia* infection and offer potential avenues for the development of new anti-*C. psittaci* strategies.

## 1 Introduction

*Chlamydia psittaci*, an obligate intracellular Gram-negative bacterium, is the etiological agent of a mild febrile respiratory zoonosis called psittacosis (Rohde et al., 2010). Birds are the major reservoirs of *C. psittaci*, and psittacosis usually occurs in persons with a history of contact with birds or poultry in either occupational settings or companion bird exposure. The clinical manifestations of human psittacosis largely present as a non-specific influenza-like illness or community-acquired pneumonia (CAP), accounting for approximately 1% of annual CAP cases (Hogerwerf et al., 2017). Over the past 20 years, several *C. psittaci* outbreaks have been reported in different countries with fatality rates of less than 1% (Mair-Jenkins et al., 2018; Yao et al., 2022).

To establish infection in host cells, *Chlamydia* must complete a unique biphasic developmental cycle where the organism alternates between the elementary body (EB) and the reticulate body (RB). EBs, the infectious form, facilitate host cell invasion through pathogen-mediated endocytosis, while RBs, though replication-competent, lack infectivity proliferation (Rucks, 2023). The infections are initiated when EBs invade host cells. After entry, EBs differentiate into RBs. During the mid-to-late infectious cycle, RBs proliferate within a specialized membrane-bound vacuole, termed the inclusion, before reverting to EBs. At the end of the infectious cycle, EBs are released by lysis of the host cells or extrusion of the inclusions and then infect neighboring cells (Omsland et al., 2012).

The obligate intracellular nature of *Chlamydia* has posed significant challenges for genetic manipulation, thereby impeding the elucidation of its pathogenic mechanisms. Consequently, studies have focused on identifying host factors contributing to the *Chlamydia* infection process. RNA interference (RNAi) screening in human cells provides a robust and straightforward genetics approach for unbiased identification of host genetic loci required for bacterial pathogenesis. Based on RNAi screening, Elwell et al. (2008) identified genes involved in heparan sulfate biosynthesis during *Chlamydia muridarum* infection in *Drosophila melanogaster* S2 cells (Elwell et al., 2008); Rosmarin et al. (2012) performed a loss-of-function genetic screen in human haploid cells and found that knockout of B3GAT3, B4GALT7, and SLC35B2 inhibited *Chlamydia trachomatis* attachment (Rosmarin et al., 2012). These findings have shed lights on revealing the mechanism by which *Chlamydia* hijacks host factors for infection.

*Chlamydia* subverts the membrane trafficking pathways of host cells to facilitate its intracellular survival, inhibiting the fusion of the inclusion with some compartments (e.g., lysosomes) while promoting fusion with others (e.g., nutrient-rich exocytic vesicles) (Elwell et al., 2016). During *Chlamydia* infection, host fusion regulators, including RAB GTPases and their effectors, SNARE proteins, and phosphoinositide lipid kinases, were recruited to inclusions and function to mediate nutrition trafficking, Golgi fragmentation, vesicular trafficking, and bacteria-host interactions. In this study, to identify human membrane trafficking factors primarily required for efficient *C. psittaci* infection, we conducted a high-throughput RNAi-based library screening with fluorescence-labeled *C. psittaci*. We pinpointed several genes that encode the coatomer complex I (COPI) subunits, affecting the *Chlamydia* proliferation process. In subsequent validation studies, we found that the knockdown (KD) of COPB1, a subunit of the COPI, induced type I interferon signaling activation, thus inhibiting the bacterial survival.

## 2 Materials and methods

### 2.1 Bacterial strains and cell culture

The human embryonic kidney epithelial cell line (HEK 293T), human cervical cancer cell line (HeLa) and human monocyte leukemia cell line (THP-1) were obtained from the American Type Culture Collection (ATCC). HEK 293T and HeLa cells were cultured in Dulbecco’s Modified Eagle’s Medium (DMEM) containing 10% fetal bovine serum (FBS) at 37°C in a 5% CO_2_ incubator, and THP-1 cells were cultured in RPMI 1640 medium supplemented with 10% FBS at 37°C in a 5% CO_2_ incubator.

The *C. psittaci* 6BC strain and GFP-expressing *C. psittaci* strain were maintained in our laboratory and propagated in HeLa cells at 37°C in a 5% CO_2_ incubator.

### 2.2 Antibodies and reagents

The primary antibodies used in this study were as follows: rabbit anti-phospho-IRF3 (Ser396) monoclonal antibody (#4947, CST), rabbit anti-phospho-TBK1 (Ser172) monoclonal antibody (#5483, CST), mouse anti-TBK1 monoclonal antibody (67211-1-Ig, Proteintech), rabbit anti-IRF3 polyclonal antibody (11312-1-AP, Proteintech), mouse anti-FLAG monoclonal antibody (F1804, Sigma-Aldrich), rabbit anti-COPA polyclonal antibody (A304-515A, ThermoFisher), mouse anti-GAPDH monoclonal antibody (60004-1-Ig, Proteintech), rabbit anti-COPB1 polyclonal antibody (K002018P, Solarbio), rabbit anti-TGN46 polyclonal antibody (13573-1-AP, Proteintech).

The following reagents were purchased from the manufacturers as noted: The silencer human membrane trafficking siRNA Library contains 426 ambion silencer siRNAs targeting 142 genes (A30139, ThermoFisher), Lipofectamine RNAiMAX reagent (13778150, ThermoFisher), human IFN-β recombinant protein (300-02BC, Peprotech), Protein A/G Sepharose FF beads (17528001, 17061801, Cytiva), C176 (HY-112906, MCE).

### 2.3 Chlamydial transformation

*Chlamydia psittaci* transformation was performed as described in a previous study (Shima et al., 2020). Briefly, pCps-Tet-mCherry plasmid was extracted from *E. coli* GM2163. PEN (1 U/mL) was used for the selection of transformed *Chlamydia*. A mixture of *C. psittaci* and pCps-Tet-mCherry were incubated in 200 μL CaCl_2_ buffer (10 mM Tris, 50 mM CaCl_2_, Ph 7.4) for 30 min at room temperature. Then, the mixed was incubated with 200 μL CaCl_2_ of trypsinised HeLa cells for a further 20 min at room temperature. A total of 100 μ L of this mixture was then added to a single well in a six well tray together with 2 mL of DMEM containing 10% FBS. Passages were performed every 2 days. GFP expression was observed by passage three. Isolation of clonal populations was achieved by the limiting dilution method, as previously described (Keb and Fields, 2020).

### 2.4 Genome-wide siRNA screen

The siRNA library was made by diluting siRNAs to 10 μM and divided into aliquots in 96-well plates and stored at −*80*°C until the time of transfection. The day before transfection, HeLa cells were seeded in 96-well plates at density of 5 × 10^4^ cells per well, for three transfection replicates and a non-target siRNA was used as a negative control (siNC). The Lipofectamine RNAiMAX reagent in serum-reduced medium Opti-MEM was added to the siRNA in each well and allowed to form siRNA-lipid complexes, which resulted in a final siRNA concentration of 60 nM.

After 24 h at 37°C, the siRNA-transfected cells were infected with GFP-expressing *C. psittaci* for 48 h at an MOI (multiplicity of infection) of 1 and the plates were incubated at 37°C with 5% CO_2_. At 48 h postinfection, the plates were imaged on an ImageXpress Micro Confocal High-Content Imaging System (Molecular Devices) using a 10 × 10 ELWD objective and the cumulative GFP fluorescence intensity was determined by High-Content automatic analysis software as a measure of *C. psittaci* proliferation. The fluorescence intensity of the GFP was initially normalized based on the confluence level per well, and subsequently analyzed as a percentage relative to the green integral intensity of the control wells containing bacteria. The fluorescence intensities of cells treated with control siRNAs (siNC) targeting no genes were directly monitored as the control. The averaged normalized values from the three technical replicates were calculated. The screen was performed in triplicates.

### 2.5 Validation of siRNA-mediated knockdown of COPI-specific target genes

To validate the knockdown effect of COPI observed in high-throughput screening results, primarily verified the silencing effect of siRNA, specific siRNAs targeting COPA and COPB1 (each target gene is represented by three different siRNAs, Table 1) were synthesized from GenePharma and transfected into HeLa cells using Lipofectamine RNAiMAX. After 48 h of transfection, cell lysates were harvested, and the protein expression levels of the target proteins as well as the mRNA transcription levels (primers shown in Table 2) were assessed using reverse transcription-quantitative PCR (RT-qPCR) or western blotting.

**TABLE 1:** Sequences of siRNA for COPA and COPB1 silence.

siRNA	Sence siRNA Sequence (5′-3′)	Antisence siRNA Sequence (5′–3′)
COPA-1	GGCAUUGACU UCCAUAAGCTT	GCUUAUGGAAGU CAAUGCCTC
COPA-2	CCUUGGAUCCU GACUAGUUTT	AACUAGUCAGGAU CCAAGGTC
COPA-3	GGGCACAACCAU UAUGUGATT	UCACAUAAUGGUU GUGCCCTG
COPB1-1	CCAACAUGGUUG AUUUAAATT	UUUAAAUCAACCA UGUUGGTG
COPB1-2	GGAUCACACUAUC AAGAAATT	UUUCUUGAUAGUGU GAUCCTG
COPB1-3	GGAUCUUCAACAUC CUAAUTT	AUUAGGAUGUUGAAGAU CCTT
*siNC	UUCUCCGAACGUGUC ACGUTT	–

*Negative control: non-specific siRNA.

**TABLE 2:** Primers for reverse transcription-quantitative PCR (RT-qPCR).

Primer	Sequence (5′–3′)
h*copb1*-F	GCGGCTGAGAACGTATGCTA
h*copb1*-R	AGCACAAAACGAATGATGGTCA
h*gapdh*-F	AACGGATTTGGTCGTATTG
h*gapdh*-R	GGAAGATGGTGATGGGATT
h*ifn*β-F	TTGTTGAGAACCTCCTGGCT
h*ifn*β-R	TGACTATGGTCCAGGCACA

A total of 24 h post-transfection, GFP-expressing *C. psittaci* was utilized to infect the transfected cells at an MOI of 1. The cell supernatant and pellets were collected at various time points and the intracellular growth and proliferation levels of *C. psittaci* were quantitatively analyzed.

### 2.6 Bacterial growth curves

THP-1 cells were seeded into 24-well plates at a density of 1 × 10^6^ cells/mL. To induce differentiation, 200 nM phorbol 12-myristate 13-acetate (PMA) was added to the culture. The differentiated THP-1 macrophages were then co-incubated with IFN-β (50 ng/mL) and infected with GFP-expressing *C. psittaci* at an MOI of 1, while cells infected without IFN-β treatment were used as controls. Similar experiments were conducted using HeLa cells. Quantitative PCR (qPCR) was employed to assess the growth and proliferation dynamics of *C. psittaci* in both groups at various time points (primers shown in Table 3).

**TABLE 3:** Primers and probes for quantitive PCR (qPCR).

Primer	Sequence (5′–3′)
Cps-F	CCCACATAGTGCCATCGAT
Cps-R	GGTTCCGCTCTCTCCTTACAAG
Cps-probe	5′6-FAM-TGCCTGTAGGGAACCCAGCTGAAC-3′-BHQ1

### 2.7 Luciferase assay

HEK 293T cells were uniformly inoculated into a 24-well cell culture plate at a density of 3 × 10^5^ cells per well. siNC and siCOPB1 were transfected into the cells at a concentration of 100 nM using Lipofectamine RNAiMAX Reagent. 24 h post-transfection, the ISRE-Luc luciferase reporter plasmid (200 ng), Renilla luciferase internal reference plasmid (20 ng), and FLAG-STING plasmid (200 ng) were co-transfected into the cells. After an additional 24 h incubation period, the luciferase activity in the total cell lysate was quantified using a dual-luciferase reporter assay kit (RG029, Beyotime).

### 2.8 Reverse transcription-quantitative PCR (rT-qPCR)

Total RNA of HEK 293T cells was extracted from the transfected cells using the RNeasy Mini Kit (QIAGEN) according to the manufacturer’s protocol, the isolated RNA was reverse-transcribed into cDNA using the TransScript one-step gDNA removal and cDNA synthesis super hybrid kit (TransGen Biotech). The relative expression levels of *ifn*β mRNA (primers shown in Table 2) in the host cells were quantified via real-time PCR with PowerUp SYBR Green Master Mix (Applied Biosystems), using GAPDH as the internal control for normalization.

### 2.9 Phosphorylation analysis via western blotting

Protein extracts of HEK 293T cells were prepared by lysing the cells with a buffer solution composed of Western and IP lysis buffer, PMSF, protease inhibitors, and phosphatase inhibitors at a ratio of 100: 1: 1: 1. The lysed samples underwent centrifugation, and the resultant supernatant was collected for subsequent SDS-PAGE protein separation, following this, proteins were transferred onto a PVDF membrane for western blotting analysis. Antibodies including anti-phospho-TBK1, anti-phospho-IRF3, anti-TBK1, and anti-IRF3 were utilized during this procedure. Finally, the western blotting data were quantitatively analyzed using ImageJ software.

### 2.10 Immunofluorescence

Cells were washed three times with phosphate-buffered saline (PBS) to remove cellular debris. Subsequently, cells were fixed with 4% paraformaldehyde for 10 min at room temperature, permeabilized with 0.1% Triton X-100 for 15 min, and blocked with 1% bovine serum albumin (BSA) in PBS for 60 min. Primary antibodies were incubated for 1 h, followed by incubation with goat anti-mouse IgG Alexa Fluor 488 and goat anti-rabbit IgG Alexa Fluor 594 secondary antibodies for 1 h in the dark, DAPI staining was performed for 5 min in the dark, each step was interspersed with three PBS washes. Finally, each well was filled with 500 μL of PBS, and imaging analysis was conducted using a confocal high-content imaging system.

### 2.11 Immunoprecipitation

Small interfering RNA transfection was performed in HEK 293T cells, the eukaryotic plasmid FLAG-STING was introduced into the cells on 24 h post-transfection. An additional 24 h period was allowed for plasmid expression before whole-cell protein extraction. The cell lysate was prepared using a mixture of Western and IP lysis buffer, protease inhibitor cocktail, and PMSF in a ratio of 100: 1: 1, cells were lysed on ice for 60 min in 450 μL of pre-chilled lysis buffer, followed by centrifugation at 10000 × *g* for 10 min at 4°C, and the supernatant was collected. The cell supernatant was combined with 100 μL of Protein A/G Sepharose FF beads and incubated at 4°C for 6 h, followed by centrifugation at 500 × *g* for 5 min at 4°C. The supernatant was retained, and 80 μL was taken as the whole-cell lysate (WCL), SDS-PAGE loading buffer (5 ×) was added to the WCL samples at a volume of 20 μL, mixed thoroughly and boiled at 100°C for 10 min, after cooling on ice, the samples were stored at −*80*°C.

## 3 Results

### 3.1 An RNAi library-based screening for human membrane trafficking factors that affect *C. psittaci* infection

**FIGURE 1:**
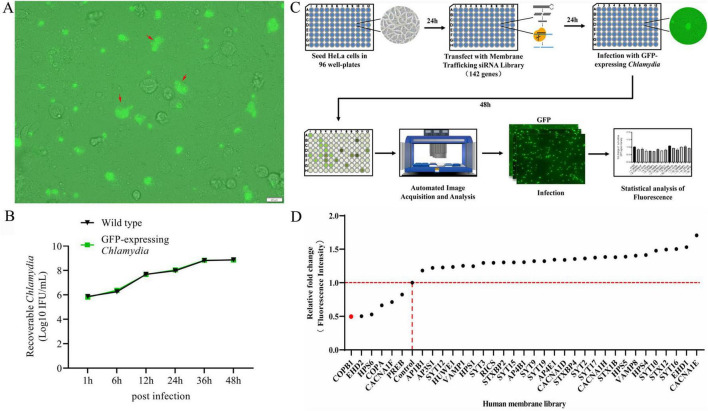
An RNA interference (RNAi) library-based screening for human membrane trafficking factors that affect *C. psittaci* infection. The pCps-Tet-mCherry plasmid shuttle vector was transformed into *C. psittaci* to constitutively expressing green fluorescent protein (GFP) (GFP-expressing *C. psittaci*) **(A)**. HeLa cells were infected with wild type *C. psittaci* and GFP-expressing *C. psittaci*, and the growth and proliferation dynamics of bacteria were assessed **(B)**. Host proteins affecting the intracellular proliferation of *C. psittaci* in HeLa cells was identified by conducting a GFP-expressing *C. psittaci* and using a human membrane trafficking siRNA library screening combined with the automated high-throughput fluorescence microscopy-based assay **(C)**. The identified human membrane trafficking factors that affected *C. psittaci* infection in HeLa cells **(D)**.

We used the pCps-Tet-mCherry plasmid shuttle vector for screening host factors involved in *C. psittaci* infection (Shima et al., 2020). pCps-Tet-mCherry contains genes encoding green fluorescent protein (GFP) and ampicillin resistance (AmpR). The plasmid was transformed into the *C. psittaci* 6BC strain for constitutive GFP expression (Figure 1A). The previous study (Shima et al., 2020) and our experimental data demonstrated that pCps-Tet-mCherry was stably retained in *C. psittaci* under selection pressure, and it did not interfere with chlamydial growth (Figure 1B).

The Human Membrane Trafficking siRNA Library (designed by Thermofisher) contains 426 siRNAs targeting 142 genes known or predicted to participate in human membrane trafficking or remodeling (Supplementary Table 1). This library is designed with an aim to understand gene expression in critical cellular functions including biosynthesis, secretion, endocytosis, lysosomal and proteasomal protein degradation, and phagocytosis. Initially, we silenced the expression of these 142 genes in HeLa cells using the Human Membrane Trafficking siRNA Library. Then 24 h later, these cells were infected with GFP-expressing *C. psittaci*. Subsequently, host cell fluorescence, which was associated with the effect of each gene-specific KD on the bacterial inclusion formation and proliferation, was monitored after 48 h postinfection using the automated high-throughput fluorescence microscopy-based assay (Figure 1C).

A set of 108 genes KD had no effect on the infection of *C. psittaci* in all three replication screens, and 28 KD were beneficial for the pathogen’s proliferation. While 6 genes KD impaired bacterial proliferation (Figure 1D), among which two top candidate genes—COPA and COPB1 encode indispensable subunits of the coatomer complex I (COPI), and hence COPB1 became the focus of our investigation.

### 3.2 COPB1-knockdown inhibits *C. psittaci* intracellular proliferation

**FIGURE 2:**
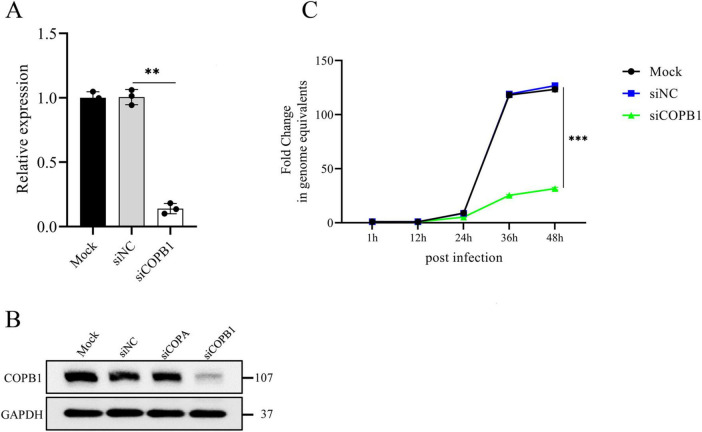
COPB1 knockdown inhibits *C. psittaci* intracellular proliferation. HeLa cells were either transfected with siRNA targeting COPB1 (siCOPB1) or control siRNAs targeting no genes (siNC), and the relative mRNA expression level of *copb1* **(A)**, the protein expression level of COPB1 **(B)** and the growth curve of *C. psittaci* in cells **(C)** were analyzed. Data are representative of three independent experiments and bars represent the mean ± SD. Statistical analysis was performed by Student’s *t*-test. **p < 0.01; ***p < 0.001.

To address how COPB1 affects *C. psittaci* intracellular proliferation, we tried to generate stable COPB1 knockout/knockdown cells several times. Consistent with a prior report (Park et al., 2019), we were unable to generate cells fully/partial deficient in COPB1. Then we used siRNA-mediated gene silencing to transiently knock down COPB1 in HeLa cells. The knockdown efficiency was assessed by RT-qPCR and western blotting. There was a significant down-regulation in the transcription of COPB1 24 h after transduction in cells transfected with siRNA targeting COPB1 (siCOPB1) (Figure 2A), and a marked reduction in COPB1 appeared (Figure 2B). To confirm the effect of COPB1 on chlamydial infection, COPB1 KD HeLa cells were then infected with *C. psittaci*, and the intracellular proliferation of *Chlamydia* was measured for 2 days. There was a significant decrease in *C. psittaci* proliferation in COPB1 KD HeLa cells as compared with non-targeting siRNA treated cells (siNC) (Figure 2C). Similar results were also obtained in COPA KD HeLa cells (Supplementary Figure 1).

### 3.3 COPB1-knockdown induced IFN-I signaling activation

COPA is a component of COPI that mediates the cargo sorting for retrograde Golgi-to-ER trafficking (Bethune and Wieland, 2018), and expression of COPA mutants activated STING pathway for inducing the expression of type I interferon (IFN, IFN-α, and IFN-β) (Mukai et al., 2021). COPA and COPB1 are both subunits of the COPI, and we reasoned that the COPB1 KD might activate the type I IFN signaling.

**FIGURE 3:**
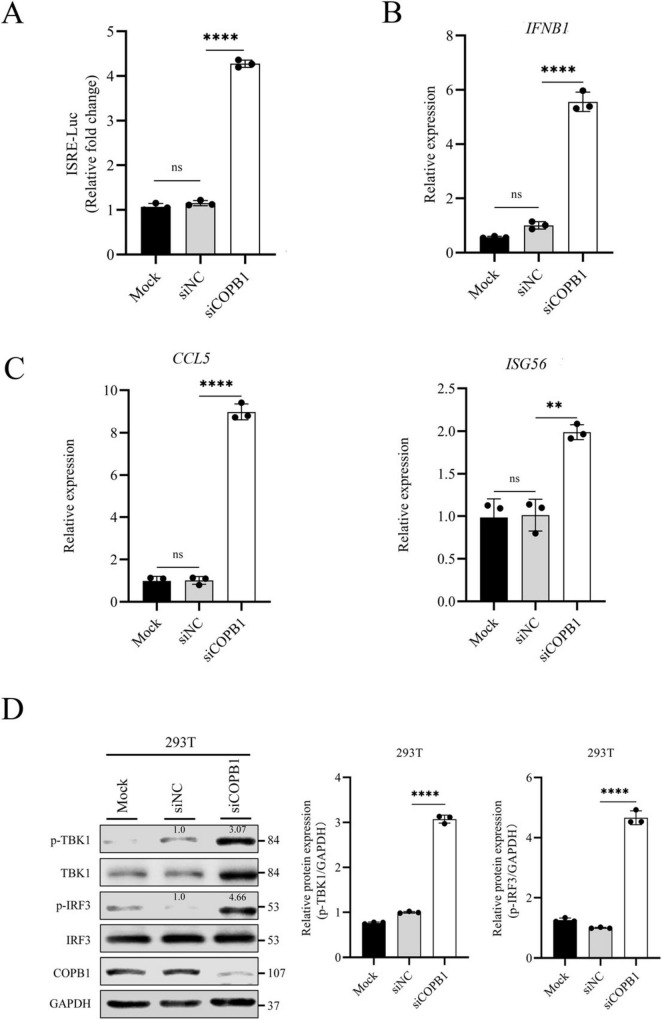
COPB1-knockdown induced IFN-I signaling activation. HEK293 cells encoding luciferase under the transcriptional control of an interferon-stimulated response element (ISRE-luciferase) were either transfected with small interfering RNA (siRNA) targeting COPB1 (siCOPB1) or control siRNAs targeting no genes (siNC), and then the luciferase activity **(A)**, levels of *IFNB* mRNA **(B)**, *CCL5* and *ISG56* mRNA **(C)**, and the phosphorylation of TBK1 and IRF3 **(D)** in the total cell lysate were measured. Data are representative of three independent experiments and bars represent the mean ± SD. Statistical analysis was performed by Student’s *t*-test or one way anova. **p < 0.01; ****p < 0.0001.

To validate this hypothesis, we examined type I IFN signaling activation in COPB1 KD cells. Human embryonic kidney (HEK293) cells, which express luciferase under the control of an interferon-stimulated response element (ISRE-luciferase), were transfected with either siCOPB1 or siNC. The luciferase activity in the total cell lysate was then measured (Cheng et al., 2023). As shown in Figure 3A, both wild-type cells and cells transfected with siNC did not activate the ISRE promoter. Surprisingly, COPB1 KD cells showed the highest activation of the ISRE promoter (Figure 3A). This indicated that COPB1 KD activates the type I IFN signaling. Levels of *IFNB* mRNA were measured to confirm that COPB1 KD activates the type I IFN signaling. Similar to ISRE-luciferase data, IFN-β expression was induced in COPB1 KD cells (Figure 3B). RT-qPCR analysis also indicated that COB1 KD promoted the transcription of downstream IFN-induced genes including *CCL5* and *ISG56* (Figure 3C).

In type I IFN signaling pathway, activated STING binds to the TRAF family member-associated NF-κB activator (TANK)–binding kinase 1 (TBK1) and inhibitor of nuclear factor κB kinase ε (IKKε), which phosphorylate IFN regulatory factor 3 (IRF3); pIRF3 then translocates to the nucleus, where it induces the expression of genes encoding IFN-β and other cytokines (Zhang et al., 2019; Jiang et al., 2020). We then examined activation of type I IFN signaling in these cells by western blotting. Consistently, COPB1 KD significantly increased the phosphorylation of TBK1 and IRF3 compared to cells transfected with siNC (Figure 3D).

Thus, our results implied that COPB1 KD-induced activation of type I IFN signaling.

### 3.4 COPB1-knockdown alters STING localization to the Golgi

**FIGURE 4:**
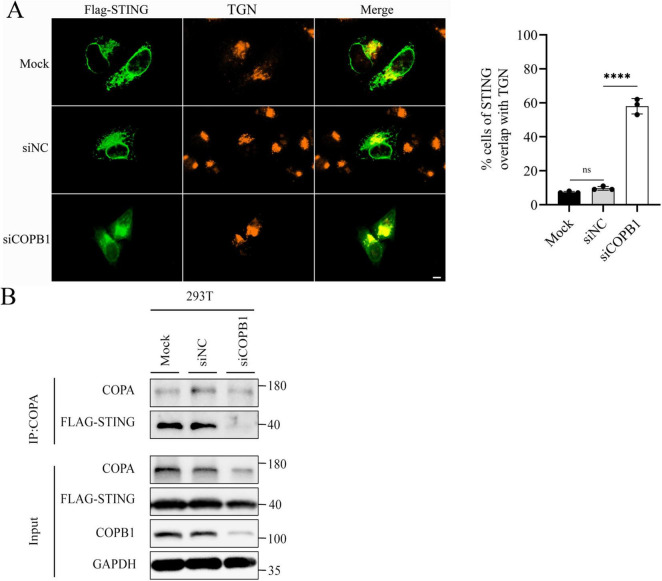
COPB1 knockdown alters STING localization to the Golgi. HEK293 cells expressing Flag-STING were either transfected with small interfering RNA (siRNA) targeting COPB1 (siCOPB1) or control siRNAs targeting no genes (siNC). Cells were fixed, permeabilized, and stained for Flag and TGN46 (a Golgi protein) (Left). The rates of STING overlap with TGN were determined by counting at least 100 cells from each sample (Right) **(A)**. Cell lysates were prepared and Flag-STING was immunoprecipitated with anti-COPA antibody **(B)**. Data are representative of three independent experiments and bars represent the mean ± SD. Statistical analysis was performed by Student’s *t*-test. ****p < 0.0001.

As part of COPI in host cells, COPB1 mediates the cargo sorting for retrograde Golgi-to-endoplasmic reticulum (ER) trafficking (Jackson, 2014). Previous studies have confirmed an interaction between STING and COPA, showing that STING is a cargo of the retrograde transport of COPI (Deng et al., 2020; Jeltema et al., 2023). We next investigated the subcellular localization of STING in host cells. In cells expressing wild-type COPB1 or siNC cells, Flag-STING distributed throughout the cytoplasm (Figure 4A). In contrast, in COPB1 KD cells, Flag-STING mostly localized with Golgi (Figure 4A). This result indicated that COPB1 KD altered the STING localization. Then we examined whether the cargo sorting of STING into COPI vesicles was impaired in COPB1 KD cells. The amount of STING in COPB1 KD cells was smaller than that with wild-type COPB1 cells or siNC cells in the co-immunoprecipitation assay (Figure 4B), further supporting that STING could not be retrieved back to the ER and accumulated in the Golgi in the absence of COPB1. These resulted suggested that the accumulation of STING in the Golgi induced by COPB1 KD activated type I IFN signaling.

### 3.5 STING induced IFN-I signaling activation inhibits *C. psittaci* intracellular proliferation

**FIGURE 5:**
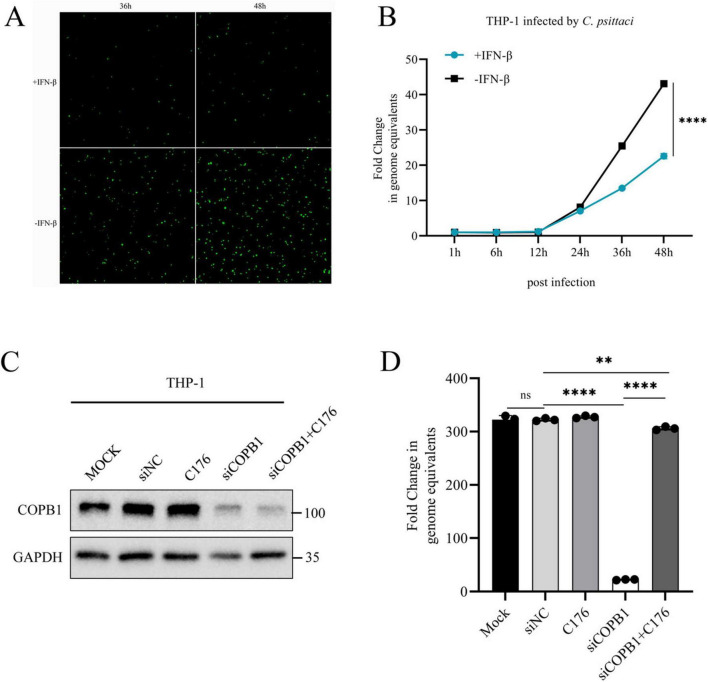
STING Induced IFN-I Signaling Activation Inhibits *C. psittaci* intracellular proliferation. THP-1 cells were pretreated with IFN-β and infected with *C. psittaci*, and then the proliferation of *C. psittaci* in cells was observed under a fluorescence microscope (magnification, × 100) **(A)**, and the growth curve of *C. psittaci* in cells was analyzed **(B)**. THP-1 cells were either transfected with siRNA targeting COPB1 (siCOPB1) or control siRNAs targeting no genes (siNC), and then these cells were treated with C-176 and infected with *C. psittaci*. The protein expression level of COPB1 **(C)** and the growth curve of *C. psittaci* in cells at 48hours postinfection **(D)** were analyzed. Data are representative of three independent experiments and bars represent the mean ± SD. Statistical analysis was performed by Student’s *t*-test or one way anova. **p < 0.01; ****p < 0.0001.

The type I IFN signaling pathway is one of the most important mechanisms of innate immunity against viral and non-viral pathogens infection. To investigate the potential impact of COPB1 KD induced IFN-I signaling activation on the intracellular proliferation of *C. psittaci*, we firstly investigated the role of type I IFN in *C. psittaci* infection. THP-1 macrophages were treated with IFN-β prior to infection, and the intracellular proliferation of *C. psittaci* was assessed over a 2 days period. A significant reduction in *C. psittaci* proliferation was observed in THP-1 cells pretreated with IFN-β compared to untreated cells (Figures 5A, B). And the same results were required in HeLa cells treated with IFN-β (Supplementary Figure 2), indicating that type I IFN restricts *C. psittaci* proliferation.

C-176 is a selective and covalent antagonist of STING, thereby inhibiting the activation of type I IFN signaling pathway (Haag et al., 2018; Sun et al., 2024). C-176 cannot reduce RIG-I or TBK1-mediated activation of type I IFN signaling pathway. The expression of COPB1 was reduced in siCOPB1 treated THP-1 cells (Figure 5C), and subsequently these cells were treated with C-176 and infected with *C. psittaci*. The result showed that the intracellular *C. psittaci* proliferation was significantly reduced in COPB1 KD cells compared with the untreated cells (Figure 5D and Supplementary Figure 3), indicating that COPB1 KD effectively inhibited the intracellular proliferation of *C. psittaci*. Furthermore, when COPB1 KD cells were pretreated with C176, the reduction of *C. psittaci* proliferation were partially restored (Figure 5D and Supplementary Figure 3), implying that the inhibition of *C. psittaci* proliferation by COPB1 KD could be rescued in by treated with C176.

Thus, these findings suggested that COPB1 KD-induced activation of type I IFN signaling inhibits *C. psittaci* intracellular proliferation.

## 4 Discussion

Genome-wide siRNA screening has emerged as a powerful approach for identifying host factors critical for pathogen replication. In the present study, we conducted a large-scale siRNA screen to systematically identify human membrane trafficking components that modulate *C. psittaci* infection in host cells. By using GFP-expressing *C. psittaci* and a human membrane trafficking siRNA library screening combined with an automated high-throughput fluorescence microscopy-based assay, we identified 34 host proteins affecting the intracellular proliferation of *C. psittaci* in HeLa cells.

A number of genome-wide screens for host factors involved in *Chlamydia* infection have been performed in recent years. Factors such as COPA, heparan sulfate, inosine-5′-monophosphate dehydrogenase (IMPDH), HIF-1α, and the Tom complex could efficiently inhibit *Chlamydia* growth *in vitro* and/or *in vivo* (Derre et al., 2007; Sharma et al., 2011; Rother et al., 2018; Park et al., 2019). These identified factors are associated with different biological processes of host cells. Membrane trafficking is fundamentally important for the normal function of host cells, mediating essential processes such as adhesion, division, nutrient uptake, immunity and cell migration (Allgood and Neunuebel, 2018). An increasing number of studies proved that membrane trafficking is crucial for chlamydial infection (Beatty, 2006; Brumell and Scidmore, 2007; Beatty, 2008; Scidmore, 2011). Here, we noticed COPA and COPB1, subunits of COPI, which are associated with non-clathrin-coated vesicles and involved in endosomal transport. These factors, revealed in our screen, have also been found in previous screenings (Derre et al., 2007). COPA could promote heparan sulfate cell surface presentation, thereby facilitating *C. trachomatis* attachment (Park et al., 2019). COPB1 was shown to be essential for Influenza A Virus (IAV) infection in siRNA-based study (Haas et al., 2023) and it was also required for the infection of RNA viruses including vesicular stomatitis virus and SARS-CoV-2, for facilitating essential steps in viral RNA synthesis and trafficking or viral assembly (Knoops et al., 2010; Panda et al., 2011).

Type I interferon are known for their antiviral activity. It is well established that type I IFN were upregulated during Chlamydial infection of host cells (Prantner et al., 2010). Conversely, they have been demonstrated to be detrimental during infection with *C. muridarum* in genital tract (Nagarajan et al., 2008) and in the lung (Qiu et al., 2008) and could inhibit *C. trachomatis* infectivity in HeLa and McCoy cells (de la Maza et al., 1985). In the present study, we demonstrated that IFN-β strongly inhibits *C. psittaci* proliferation in both THP-1 and HeLa cells. We also found that COPB1 knockdown in 293T cells triggered the activation of type I IFN signaling, suggesting that COPB1 KD induces type I IFN activation, which in turn inhibits *C. psittaci* intracellular proliferation.

In type I IFN signaling way, the trafficking of STING, a key mediator of responses to cyclic dinucleotides, RNA and cytosolic DNA, from the ER to Golgi is critical to activate the downstream signaling cascade (Mukai et al., 2016) and STING is then subjected to palmitoylation and activates TBK1 at TGN (Jeltema et al., 2023). In contrast, robust downstream type I IFN pathway activation is prevented by the retrieved back of STING to the ER by the COPI-mediated retrograde transport (Rivara and Ablasser, 2020; Taylor et al., 2023). In this study, we found that STING could not be retrieved back to the ER and accumulated in the Golgi in the absence of COPB1 (Figure 4), leading to type I IFN signaling activation. These results suggested that homeostatic STING trafficking was disturbed in the absence of COPB1, consistent with the results that spontaneous cGAS/STING signaling is activated by general deficiency in COPI-mediated retrograde transport (Di Marco et al., 2020; Steiner et al., 2022). As a specific inhibitor of STING, C176 can significantly reduce STING mediated IFN β reporter gene activity. In the present study, the reduction of *Chlamydia* proliferation caused by COPB1 KD in host cells could be partially recued, one explain is that in addition to the retrograde transport of STING, COPI could also promote heparan sulfate cell surface presentation to facilitating *Chlamydia* attachment (Park et al., 2019).

In summary, this study’s systematic exploration of host membrane trafficking factors implicated in *C. psittaci* infection has significantly advanced our understanding of *Chlamydia* pathogenesis. These findings not only elucidate critical host-pathogen interactions but also pave the way for the development of innovative host-directed therapeutic strategies against chlamydial infections.

## Data Availability

The original contributions presented in this study are included in this article/Supplementary material, further inquiries can be directed to the corresponding authors.
