# Supplementary figures and images for: COPB1-knockdown induced type I interferon signaling activation inhibits Chlamydia psittaci intracellular proliferation
Source: Front Microbiol. 2025 Mar 6;16:1566239. doi: 10.3389/fmicb.2025.1566239 (PMC11922848; doi:10.3389/fmicb.2025.1566239)

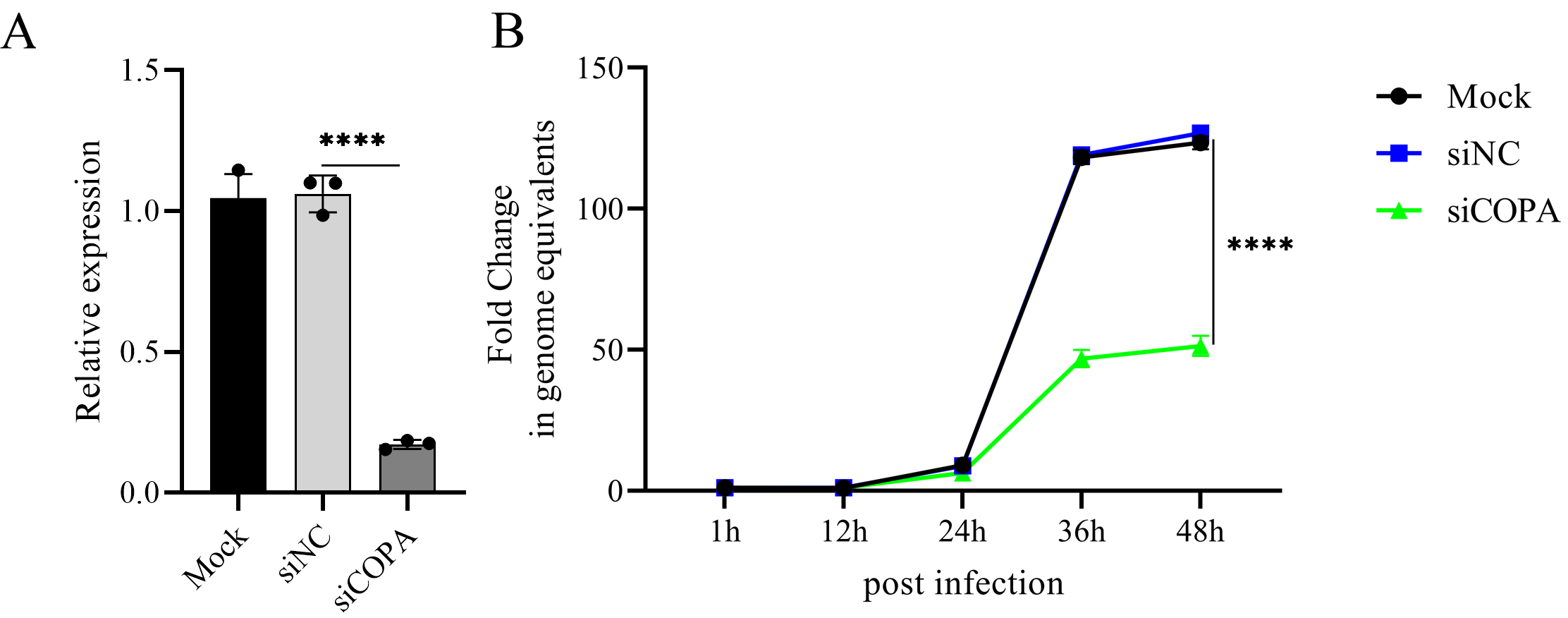

Supplement: Supplementary file 2 [file Image_1.tif]

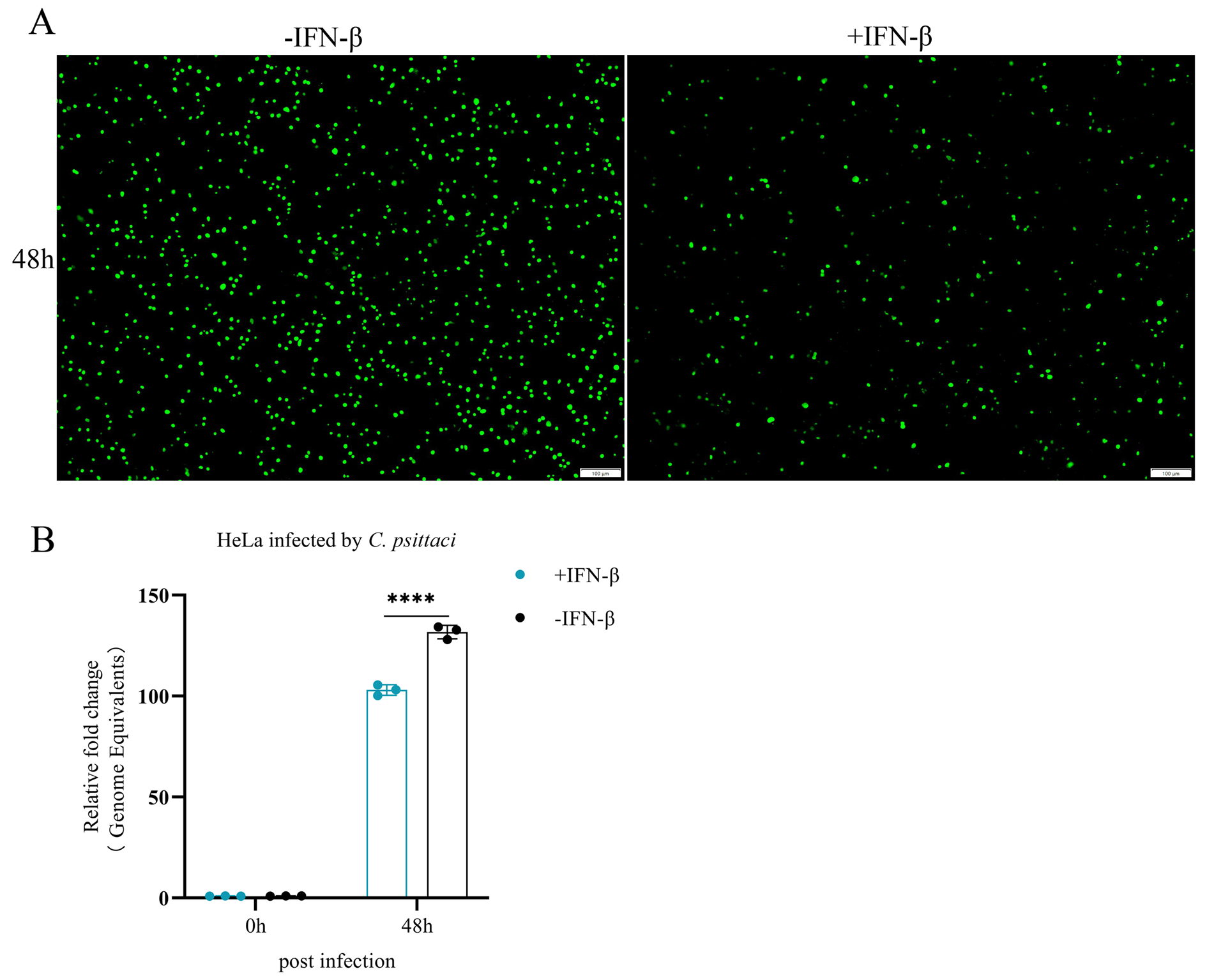

Supplement: Supplementary file 3 [file Image_2.tif]

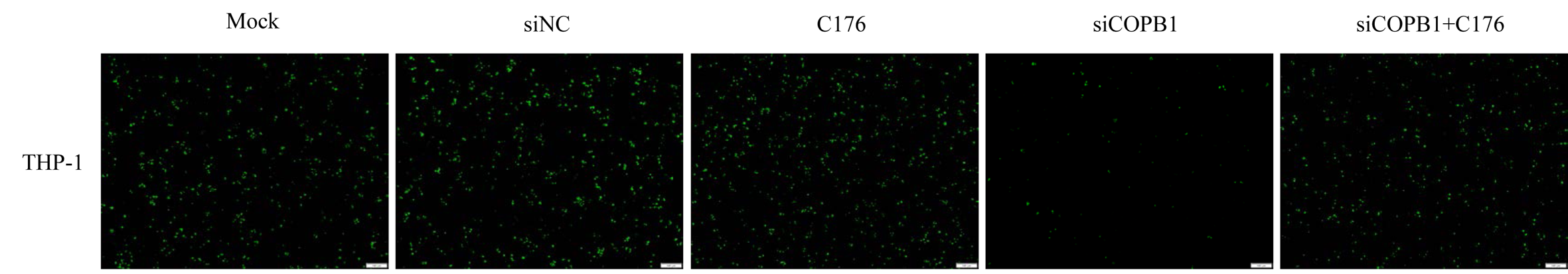

Supplement: Supplementary file 4 [file Image_3.tif]
